# Estimation with Uncertainty via Conditional Generative Adversarial Networks

**DOI:** 10.3390/s21186194

**Published:** 2021-09-15

**Authors:** Minhyeok Lee, Junhee Seok

**Affiliations:** 1 School of Electrical & Electronics Engineering, Chung-Ang University, Seoul 06974, Korea; mlee@cau.ac.kr; 2 School of Electrical Engineering, Korea University, Seoul 02841, Korea

**Keywords:** generative adversarial network, deep learning, adversarial learning, probability estimation, risk estimation, portfolio management

## Abstract

Conventional predictive Artificial Neural Networks (ANNs) commonly employ deterministic weight matrices; therefore, their prediction is a point estimate. Such a deterministic nature in ANNs causes the limitations of using ANNs for medical diagnosis, law problems, and portfolio management in which not only discovering the prediction but also the uncertainty of the prediction is essentially required. In order to address such a problem, we propose a predictive probabilistic neural network model, which corresponds to a different manner of using the generator in the conditional Generative Adversarial Network (cGAN) that has been routinely used for conditional sample generation. By reversing the input and output of ordinary cGAN, the model can be successfully used as a predictive model; moreover, the model is robust against noises since adversarial training is employed. In addition, to measure the uncertainty of predictions, we introduce the entropy and relative entropy for regression problems and classification problems, respectively. The proposed framework is applied to stock market data and an image classification task. As a result, the proposed framework shows superior estimation performance, especially on noisy data; moreover, it is demonstrated that the proposed framework can properly estimate the uncertainty of predictions.

## 1. Introduction

Conventional predictive Artificial Neural Network (ANN) models commonly operate with a feed-forward framework using deterministic weight matrices as the network weight parameters [1,2,3]. Specifically, the estimation of ANNs is conducted with matrix operations between given samples and the trained network parameters with non-linear activation functions.

While outstanding progress has been made in ANNs in recent years [4,5] and ANNs are widely used for many practical applications [6,7,8,9,10], conventional predictive ANN models have an obvious limitation since their estimation corresponds to a point estimate. Such a limitation causes the restrictions of using ANN for medical diagnosis, law problems, and portfolio management, where the risk of the predictions is also essential in practice. The conventional ANN models produce the same form of predictions even if the predictions are very uncertain, and such uncertain prediction results cannot be distinguished from confident and regular predictions.

In short, conventional ANN models cannot say ‘I don’t know’, and it corresponds to a type of overfitting since overfitting stands for failure to estimate untrained data precisely due to excessive precision for training data resulting from high flexibility of the model. For instance, the models attempt to make a confident prediction for an outlier or even complete noise data of which predictions are meaningless and impossible. In such a framework, it is not clear how much the models are sure on their predictions.

To handle such a problem, a probabilistic approach of ANNs, called Bayesian Neural Networks (BNNs), has been introduced [11,12,13]. In BNNs, values of the network weight parameters are not fixed, and instead obtained by a sampling process from certain distributions. Therefore, the prediction of BNN for a given sample differs in each operation. Integrated with the Monte Carlo method in which many different predictions are made for a given sample, the prediction of BNNs also forms a distribution of which variance can represent the risk and uncertainty of the predictions.

However, the training of BNNs is not straightforward since the Monte Carlo method is employed for the training process as well [11]. In the training process, the network parameters are sampled from posterior distributions of the network parameters, and gradients are calculated and back-propagated for the sampled parameters. Therefore, such randomness intrinsic in the training process hinders fast training and convergence of BNNs. Furthermore, the training of deep BNNs is also not straightforward due to such a problem.

As another probabilistic neural network model that can produce a form of distributions as its outputs, Generative Adversarial Networks (GANs) have shown superior performance for sample generation [14,15,16,17,18]. Generally, GANs learn the sample distribution of a certain dataset in order to produce synthetic but realistic samples from input noises by mapping features intrinsic in the dataset onto the input noise space. While typical GAN models produce random samples, conditional variants of GANs (cGANs) have been introduced to generate the desired samples and have shown fine results to produce samples by using conditional inputs [19,20,21,22]. Moreover, by taking advantage of such a innovative framework of GAN, the modified GAN models have been introduced in many applications [23,24,25,26,27,28,29].

Basically, this paper addresses the following question that has arisen from the characteristic of cGANs: Since cGANs can learn the probability distribution of samples, i.e., Pr(X|Y,Z), is it possible to learn the probability distribution of labels, i.e., Pr(Y|X,Z), by reversing the inputs and outputs of the cGANs? If it is possible, we can utilize cGANs as a predictive probabilistic neural network model, similar function to BNNs. Moreover, such a model can solve the problems in BNNs since deep architectures can be employed for GANs, and their training is relatively simple compared to BNNs. However, such an issue has not yet been studied extensively.

In this paper, we propose an adversarial learning framework for utilizing the generator in cGANs as a predictive deep learning model with uncertainty. Since the outputs of the proposed model are a form of distribution, the uncertainty of predictions can be represented as the variance of the distribution. Furthermore, in order to measure and quantify the uncertainty of estimations, we introduce the entropy and relative entropy for regression problems and classification problems, respectively.

## 2. Background

### 2.1. Problem Description

Let $X\in R^{p}$ be a sample and $Y\left(X\right)\in R^{q}$ be labels for the sample. A predictive ANN model estimates the labels for a given sample as follows:

(1) $${\hat{Y}}_{M,\vartheta_{A}}\left(X\right)=M\left(X;\vartheta_{A}\right),$$

where $M:R^{p}\mapsto R^{q}$ is an ordinary ANN structure, and $\vartheta_{A}$ denotes a set of weight parameters of the model A. Throughout the paper, we use the same notation $M_{\left(\cdot \right)}$ when an ANN structure is used, and $M_{\left(\cdot \right)}$ is used to indicate a general method that does not use ANNs.

However, such predictions correspond to point estimates and it is not clear how much the model is certain of the predictions. For instance, given a complete noise $X_{N}∼N\left(0,I\left(p\right)\right)$, it is even possible to predict a label for the noise, i.e., ${\hat{Y}}_{M,\theta }\left(X_{N}\right)$; obviously, such a point estimate is the wrong answer for noises, and one of the correct answer might be ‘I don’t know’, which is impossible to be represented in conventional ANNs.

In this paper, we aim to solve this problem by conducting the prediction in a probabilistic manner:

(2) $$\mathrm{Pr}\left(Y_{1}\left(X\right),\ldots ,Y_{q}\left(X\right)\right)=M_{P}\left(X;\vartheta_{P}\right),$$

where $M_{P}:R^{p}\mapsto D$ is a probabilistic ANN model, $Y_{j}$ denotes a variable for possible values of *j*th element of $Y\left(X\right)$, and j∈{1,…,q}. From such probability distributions of predictions, not only the point estimation but also the uncertainty of the prediction can be calculated as follows:

(3) $$\begin{array}{c} \left\{{\hat{Y}}_{M_{C},M_{P},\vartheta_{P}}\left(X\right),\eta \left({\hat{Y}}_{M_{C},M_{P},\vartheta_{P}}\left(X\right)\right)\right\}=M_{C}\left(\mathrm{Pr}\left(Y_{1}\left(X\right),\ldots ,Y_{q}\left(X\right)\right);\theta_{C}\right), \end{array}$$

where $M_{C}:D\mapsto R^{2}$ is a method for point estimation using the estimated distributions; therefore, ${\hat{Y}}_{M_{C}}$ can be the mean or median of the estimated distributions, $\theta_{C}$ is a set of parameters for $M_{C}$, and η denotes a value describing the uncertainty measure of the point estimation.

### 2.2. Stochastic Weights for Neural Networks

In order to construct a probabilistic neural network model shown in (2), BNNs using stochastic weights have been introduced [11,30,31,32]. In BNNs, each weight has a probability distribution, and a value of the weight is sampled from the probability distribution each time the model make an inference. Then, by using the Monte Carlo method over the distribution, the prediction of BNNs can be a form of distribution as follows:

(4) $${\hat{Y}}_{M_{B},\vartheta_{B}\left(i\right)}\left(X\right)=M_{B}\left(X;\vartheta_{B}\left(i\right)\right),$$

(5) $$\begin{array}{c} \mathrm{Pr}\left(Y_{1}\left(X\right),\ldots ,Y_{q}\left(X\right)\right)=M_{D}\left(\left\{{\hat{Y}}_{M_{B},\vartheta_{B}\left(1\right)}\left(X\right),\ldots ,{\hat{Y}}_{M_{B},\vartheta_{B}\left(k\right)}\left(X\right)\right\};\theta_{D}\right), \end{array}$$

where $M_{B}:R^{p}\mapsto R^{q}$ is a BNN architecture, *i* denotes an index for the weight sampling, *k* denotes the number of sampling, $\vartheta_{B}\left(i\right)∼P_{\vartheta_{B}}$ denotes the *i*th sampled weights from the weight distributions, and $M_{D}:R^{k}\mapsto D$ and $\theta_{D}$ are a method for the probability density estimation and a set of parameters for the method, respectively.

### 2.3. Generative Adversarial Networks and Their Conditional Variants

GANs are probabilistic neural network models in common with BNNs; however, in contrast to BNNs, GANs use deterministic weight parameters, and, instead, employ a stochastic noise vector as their input for representing latent features in a dataset [33,34,35,36,37]. The training of GANs is performed in an adversarial manner in which a discriminator and a generator play a game to distinguish and deceive each other. Generally, GANs are used to learn sample distributions; therefore, by varying the noise vector with the Monte Carlo method, the output of the generator is a synthetic sample:

(6) $${\hat{X}}_{M_{G},\vartheta_{G}}\left(Z\left(i\right)\right)=M_{G}\left(Z\left(i\right);\vartheta_{G}\right),$$

where $M_{G}$ is a generator in GANs, $Z\left(i\right)∼P_{Z}$ is a noise vector, and $\hat{X}$ is a generated synthetic sample.

However, since it is not clear which noise variable is related to which feature, producing desired samples is challenging in ordinary GANs. In order to address such a problem, conditional variants of GANs have been studied. Conditional GAN (cGAN), one of the most popular of the conditional variants, is extensively used to generate conditional samples [19,38,39,40]. cGAN uses labels as another input for the following generator:

(7) $${\hat{X}}_{M_{cG},\vartheta_{cG}}\left(Z\left(i\right),Y\right)=M_{cG}\left(Z\left(i\right),Y;\vartheta_{cG}\right),$$

where $M_{cG}$ is a generator in cGANs, and Y is a desired condition, which is basically the same form as $Y\left(X\right)$.

## 3. Methods

### 3.1. Conditional Generative Adversarial Networks as a Prediction Model

In this paper, we proposed a new framework to use the generator in cGAN as a predictive model while the existing cGAN is routinely employed for sample generation. By simply reversing the output and the conditional input in cGAN and using the same form of network architectures, the model can successfully be used as a probabilistic predictive neural network model, which has the same function as BNNs:

(8) $$\begin{array}{c} {\hat{Y}}_{M_{cG},\vartheta_{cG}}\left(Z\left(i\right),M_{F}\left(X;\vartheta_{F}\right)\right)=M_{cG}\left(Z\left(i\right),M_{F}\left(X;\vartheta_{F}\right);\vartheta_{cG}\right) \end{array}$$

where $M_{F}:R^{p}\mapsto R^{u}$ is a feature network that extracts *u*-dimensional features from samples. Therefore, ${\hat{Y}}_{M_{cG}}$ corresponds to one of the prediction results using cGAN; we can use the sample X instead of the feature network if the dimension of the sample space, i.e., *p*, is low. Such a modification simply changes the input and output in (7).

By sampling different noise vectors $Z\left(i\right)$, a probability distribution of predictions can be obtained in a similar manner with BNNs as described in (5). Such a sampling process is the same with that of ordinary GAN in (6). Therefore, the inference process of the proposed model is similar to ordinary GAN using $Z\left(i\right)∼P_{Z}$. However, while each noise vector can produce a synthetic sample in the ordinary GAN, predictions using the proposed method are conducted with a bunch of noise vectors so that a form of distribution is constructed as follows.

(9) $$\begin{array}{c} \mathrm{Pr}\left(Y_{1}\left(X\right),\ldots ,Y_{q}\left(X\right)\right)\\ =M_{D}\left(\left\{{\hat{Y}}_{M_{cG},\vartheta_{cG}}\left(Z\left(1\right),M_{F}\left(X;\vartheta_{F}\right)\right),\ldots ,\right.\right.\left.\left.{\hat{Y}}_{M_{cG},\vartheta_{cG}}\left(Z\left(q\right),M_{F}\left(X;\vartheta_{F}\right)\right)\right\};\theta_{D}\right). \end{array}$$

In the training of GAN structures, the generator is trained in an adversarial manner to deceive the discriminator; therefore, the discriminator is required to be set in order to train $M_{cG}$. In this paper, the projection discriminator [19], which shows superior performance compared to simple concatenation, is employed for the training of the generator. The architecture of the projection discriminator is as follows:

(10) $$\begin{array}{c} M_{Dis}\left(X,Y\left(X\right);\vartheta_{Dis}\right)=W_{o}\cdot M_{\varphi }\left(Y\left(X\right);\vartheta_{\varphi }\right)+M_{F}{\left(X;\vartheta_{F}\right)}^{T}\cdot M_{\varphi }\left(Y\left(X\right);\vartheta_{\varphi }\right), \end{array}$$

where $M_{Dis}:\left\{R^{p},R^{q}\right\}\mapsto R$ denotes the projection discriminator, $W_{o}\in R^{1\times u}$ is a weight matrix for the output layer of the discriminator, $\vartheta_{Dis}=\left\{W_{o}\right\}\cup \vartheta_{\varphi }$, and $M_{\varphi }:R^{q}\mapsto R^{u}$ is an ANN structure with an output dimension of *u*.

In order to solve a classification problem with the proposed framework, where the input of the discriminator $Y\left(X\right)$ is a one-hot vector, the $M_{\varphi }$ can be replaced by a matrix as follows.

**Proposition** **1.** *The $M_{\varphi }$ is equivalent to $Y\left(X\right)\cdot W_{\varphi }$, where $W_{\varphi }\in R^{q\times u}$ if $Y\left(X\right)$ is a one-hot vector.*

**Proof.**  For every one-hot vector $Y_{1}\left(X\right)\;=\;\left[1,0,\ldots ,0\right],$ …, $Y_{q}\left(X\right)\;=\;\left[0,0,\ldots ,1\right]$, every possible output of $M_{\varphi }$, i.e., $M_{\varphi }\left(Y_{1}\left(X\right);\vartheta_{\varphi }\right),$ …, $M_{\varphi }\left(Y_{q}\left(X\right);\vartheta_{\varphi }\right)$, is equivalent to $Y_{1}\left(X\right)\cdot W_{\varphi },\ldots ,Y_{q}\left(X\right)\cdot W_{\varphi }$ since there exists a matrix $W_{\varphi }$ that satisfies $W_{\varphi }=$ $\left[M_{\varphi }\left(Y_{1}\left(X\right);\vartheta_{\varphi }\right),\right.$ …, ${\left.M_{\varphi }\left(Y_{q}\left(X\right);\vartheta_{\varphi }\right)\right]}^{T}$. □

Therefore, for classification problems, (10) can be simplified as the following.

(11) $$\begin{array}{c} M_{Dis}\left(X,Y\left(X\right);\vartheta_{Dis}\right)=W_{o}\cdot Y\left(X\right)\cdot W_{\varphi }+M_{F}{\left(X;\vartheta_{F}\right)}^{T}\cdot Y\left(X\right)\cdot W_{\varphi }. \end{array}$$

Hence, throughout the paper, we use (10) and (11) for regression problems and classification problems, respectively.

### 3.2. Entropy to Measure the Uncertainty of Predictions

We introduce entropy metrics to measure and quantify the uncertainty of predictions from cGANs. While the variance of estimated distribution can be used to represent uncertainty if the distribution follows a normal distribution, we employ the entropy in this paper since we cannot reject the possibility that the distribution does not follow a normal distribution. For regression problems, the regular entropy of the estimated distribution in (9) is employed, which can be calculated as follows:

(12) $$H\left(Y_{j}\right)=-\sum_{k}\mathrm{Pr}\left(Y_{j,k}\right)\cdot \log\left(\mathrm{Pr}\left(Y_{j,k}\right)\right),$$

where $\mathrm{Pr}\left(Y_{j,k}\right)$ is the probability of *k*th element of $Y_{j}$. Notice that j=1,…,q, so that the entropy is calculated for each target variable.

On the other hand, for classification problems, the relative entropy, also known as the Kullback–Leibler divergence, is used instead of the ordinary entropy since the difference in distributions between the predicted class and the other classes can represent the certainty of the point prediction. If a prediction is certain, the distribution of predicted class has low variance, and its distance from the other distributions would be far; such variance and distance can be comprehensively represented by relative entropy. Let $l_{X}$ be an index of the predicted class given a sample X, for example, $l_{X}={\mathrm{argmax}}_{j}\sum_{i}{\hat{Y}}_{j,M_{cG},\vartheta_{cG}}\left(Z\left(i\right),M_{F}\left(X;\vartheta_{F}\right)\right)$, if we use the average as the point estimation of each class. However, the relative entropy is asymmetric, which can hardly be used as a metric for uncertainty. Therefore, we use the sum of two relative entropy measures by using different orders of distributions in order to make the measure symmetric.

(13) $$\begin{array}{c} D_{KL}\left(D_{j=l_{X}}\|D_{j\neq l_{X}}\right)+D_{KL}\left(D_{j\neq l_{X}}\|D_{j=l_{X}}\right)\\ =\sum_{k}{\mathrm{Pr}}_{j=l_{X}}\left(Y_{j,k}\right)\cdot \log\left(\frac{{\mathrm{Pr}}_{j=l_{X}}\left(Y_{j,k}\right)}{{\mathrm{Pr}}_{j\neq l_{X}}\left(Y_{j,k}\right)}\right)\\ +\sum_{k}^{}{\mathrm{Pr}}_{j\neq l_{X}}\left(Y_{j,k}\right)\cdot \log\left(\frac{{\mathrm{Pr}}_{j\neq l_{X}}\left(Y_{j,k}\right)}{{\mathrm{Pr}}_{j=l_{X}}\left(Y_{j,k}\right)}\right). \end{array}$$

By employing these entropies, we can measure the uncertainty of predictions for regression problems and classification problems as follows.

(14) $$\eta \left(\hat{Y}\right):=\{\begin{array}{cc} \left\{H\left(Y_{1}\right),\ldots ,H\left(Y_{q}\right)\right\}; & \mathrm{for}\;\mathrm{regression},\\ \begin{array}{c} c-D_{KL}\left(D_{j=l_{X}}\|D_{j\neq l_{X}}\right)\\ -D_{KL}\left(D_{j\neq l_{X}}\|D_{j=l_{X}}\right) \end{array}; & \mathrm{for}\;\mathrm{classification}. \end{array}$$

Notice that $-D_{KL}$ is used to describe the uncertainty since the ordinary relative entropy represents the difference in class distributions; therefore, the minus relative entropy indicates similarity between the predicted class distribution and the other class distributions, which corresponds to uncertainty. In contrast, the ordinary entropy measures a type of variance of distributions; thereby, a high value of the entropy indicates the uncertainty of predictions.

For classification problems, however, ordinary ANN models have a sort of uncertainty measure of which the function is similar to the proposed uncertainty measure. The softmax function is commonly employed for the last layer of ANN classifiers, and the outputs of the function provide a kind of probability for each class. Therefore, $\log{\left({\hat{Y}}_{M,\vartheta_{A}}\left(X\right)\right)}_{l_{X}}$, the cross-entropy loss for the softmax function, can indicate the uncertainty of prediction. However, there exists overfitting in ordinary ANN models, as described in the previous section; therefore, this measure becomes imprecise. We will compare the performance between the proposed uncertainty measure and this existing method in Section 4.

### 3.3. Comparison to Related Works

**Figure 1:**
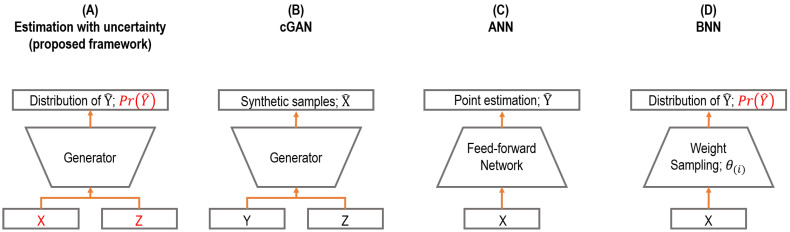
Comparison to related works. (**A**) cGAN as a prediction model (the proposed framework); (**B**) ordinary cGAN for sample generation; (**C**) artificial neural networks for prediction; (**D**) Bayesian neural networks.

In this section, we compare the proposed framework with related prior works. The key differences are summarized and illustrated in Figure 1.

**Comparison to ordinary cGANs.** The conventional generator in cGAN is used for synthetic sample generation [19]. Ordinary cGAN learns the conditional sample distribution, i.e., $\mathrm{Pr}\left(X|Y,Z\right)$, and then produces synthetic samples integrated with the Monte Carlo method over the noise vector Z. In contrast, we used cGAN as a prediction model where the model learns the target distribution, i.e., $\mathrm{Pr}\left(Y\right)$, and performs predictions as a form of distributions, i.e., $\mathrm{Pr}\left(Y|X,Z\right)$, with a stochastic input Z given a sample X as the conditional input of cGAN. In short, the neural network architectures of cGANs in both studies are basically identical, while the proposed framework corresponds to reversing the input and output in the typical use of cGAN.

**Comparison to ANNs and BNNs.** ANN has a limitation to express the uncertainty of predictions, as described in the previous sections. The output of an ANN is a point estimate, while the output of the proposed framework is a distribution that can represent the uncertainty of predictions, which can handle such a limitation. Likewise, BNN also produces predictions as a form of distribution, which is similar to the proposed framework; however, BNN uses stochastic weights to perform such work, which generally hinders the convergence in training and construction of deep neural network architecture. In contrast, the proposed framework uses deterministic weights and stochastic inputs instead. In addition, the training process is also different, where an adversarial training manner with a discriminator is employed for the proposed framework, which can avoid overfitting resulting from the high complexity of neural network architectures.

## 4. Results

### 4.1. The Prediction of Stock Prices with the Uncertainty Measure of the Prediction

Stock market prediction is one of the most specific problems where the predictions of returns and the uncertainties of the prediction are comprehensively required in practice. In modern portfolio theory [41,42,43,44], both expected returns and risks of a portfolio must be calculated for the selection of a portfolio; the prediction of the expected returns and the risks exactly corresponds to the estimation with uncertainty, the aim of the proposed framework.

We apply the proposed framework to NASDAQ-100 Future Index data. The model is trained with returns of the past 30 days as the input and the 5 day return as the target. The 5 day return is the change in prices over 5 days, calculated by $\left(P_{t+5}-P_{t}\right)/P_{t}$, where $P_{t}$ is the price at time *t*. For the training set, the close price index from January 2001 to December 2015 are used; the data from January 2016 to April 2019 are employed for the test set. For the stability of the data, the price data are preprocessed to the returns. The preprocessing process for the price data is provided in Section E in the Supplementary Materials.

**Figure 2:**
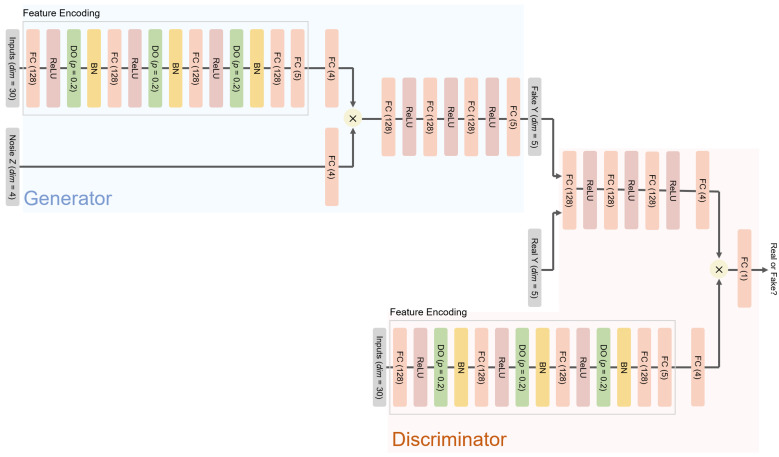
Neural network architectures of the proposed model (cGAN-UC-3). FC (*n*) indicates a fully connected layer with *n* nodes. DO (p=r) indicates a dropout layer with a dropout probability of *r*. BN represents the batch normalization. For other models, i.e., cGAN-UC-*k*, the numbers of fully connected layers, i.e., FC (128), in the generator and discriminator change to *k*.

The hinge loss and Wasserstein distance are employed for fine training of cGAN, according to recent studies of cGAN [19,45]. For probabilistic models that produce a form of distributions as their output, the mode is employed for the point estimation. The ANN and cGAN-UC, that is cGAN for the prediction with uncertainty, i.e., the proposed framework, are trained over 2000 epochs; in contrast, BNN models are trained over 5000 epochs, but the 5 layer BNN fails to converge within the training process, which demonstrates the difficulty of the training of stochastic weights in BNNs, as described previously. The architectures of the models used in this experiment are provided in Figure 2 and Section C.1 and C.4 in the Supplementary Materials.

The models are comprehensively evaluated by the prediction performance of returns and whether the estimated uncertainty is actually correlated with prediction errors, which means the risk of predictions can properly be measured. Table 1 shows correlation coefficients in the test set. The deterministic models show similar prediction performances, whereas cGAN-UCs show superior performance compared to the deterministic models as well as BNNs. The 5-layer cGAN-UC demonstrates the best prediction performance, while the uncertainty estimation is more precise in the 7-layer model. Such a resulting difference between cGAN-UG-5 and cGAN-UG-7 is from the number of layers in each model, which can differ by the hyper-parameter optimization. Moreover, we conjecture that the performance gain of the proposed model results from the adversarial learning process of GAN that can assist in learning the true distribution of noisy data and avoiding the overfitting inherent in the complex neural network architectures, which should be studied further for future work.

Moreover, it is demonstrated that the estimated uncertainty and prediction errors are correlated, which signifies that the predictions with high uncertainty have a high possibility of being wrong. While the predictions of returns by both the deterministic models and the probabilistic models are not very successful, where the correlation coefficients are below 0.1 due to the chaotic nature of the stock market, the proposed framework demonstrates that the uncertainty of the predictions can be measured. Such a result indicates a possibility to utilize the proposed uncertainty measure for risk management of portfolios using stock market predictions.

**Figure 3:**
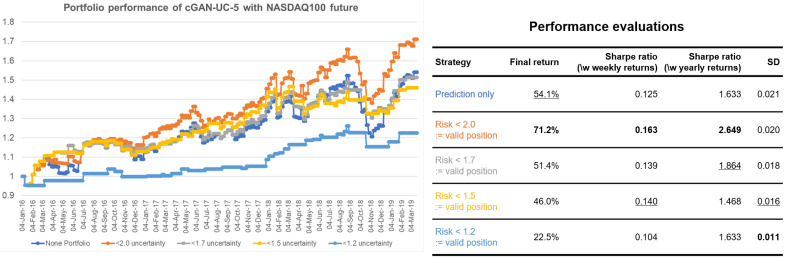
Portfolio performance with different strategies using cGAN-UC. Dark blue indicates a conventional strategy using only the prediction of returns while the others represent the strategies using the both predictions and estimated uncertainty. SD indicates the standard deviation of weekly returns. In each evaluation metric, the best performance is bold, and the second best is underlined.

Figure 3 is an example of using the estimated uncertainty for portfolio management. For evaluation metrics, final returns and Sharpe ratio of strategies are used. While the final return of strategy can measure performance, low volatility of strategy is also crucial for a financial portfolio. The Sharpe ratio can measure both returns and volatility of a strategy, which is calculated as follows:

(15) $$\begin{array}{c} SR_{A}=\frac{E\left[R_{A}-R_{f}\right]}{SD\left(R_{A}\right)} \end{array}$$

where $SR_{A}$ represents the Sharpe ratio of strategy A, SD indicates the standard deviation, $R_{f}$ is the return of a risk-free portfolio which is generally a national bond interest rate, and $R_{A}$ is the return over a specific period of time, which is calculated as follows:

(16) $$\begin{array}{c} R_{A}=\sum_{a\in U}\left[\frac{P_{a,t+k}-P_{a,t}}{P_{a,t}}\times W_{a,A}\right] \end{array}$$

where $P_{a,t}$ is the price of an asset *a* at time t, *U* is the investable universe, *k* is the period of time, and $W_{a,A}$ represents weights on the asset *a* in the strategy A such that $\Sigma_{a\in U}W_{a,A}=100\%$.

When only the predictions of returns are given, the simplest strategy that uses the predictions is taking a long position if the prediction is positive and taking a short position when it is otherwise. The portfolio performance of the simplest strategy using only the prediction results of cGAN-UC is represented with dark blue in Figure 3. We can further enhance the performance by using the estimated uncertainty (risk), along with the predictions. The other strategies employ the uncertainty by introducing a neutral position if the uncertainty is above certain thresholds; such an approach signifies that we do not take a risk for uncertain predictions. The orange strategy in the figure considers predictions with >2.0 uncertainty as invalid predictions; thus, we take neutral position in such cases. By using the strategy, the final return increases by 17.1% points, and the standard deviation of the portfolio simultaneously decreases, compared to the conventional strategy. Furthermore, interestingly, the standard deviation of the strategy with <1.7 uncertainty (gray) considerably decreases while the final returns of the strategies are similar to that of conventional strategy. Such a possibility of performance enhancement is another strong evidence that the estimated uncertainty is effective.

### 4.2. Image Classification with Uncertainty

In this section, the proposed framework is applied to an image classification task with the CIFAR-10 dataset. The CIFAR-10 dataset consists of 32×32 dimensional images, which contain 50,000 training samples and 10,000 test samples in 10 different categories. Unlike regression tasks, for classification tasks, deterministic models can estimate the uncertainty of predictions by using the probability of point estimates as the certainty of predictions since a softmax function is used for the last layer of the network in general. For instance, if a prediction of a deterministic model is ‘Car’ with a 98% probability, the uncertainty can be estimated by 2%. In this manner, for the uncertainty measure of deterministic models, the log of the probability of point estimates, i.e., the cross-entropy classification loss, is employed as a conventional method.

For the comparison between a deterministic model and cGAN-UC, densely the connected convolutional network (DenseNet), which generally shows fine performances for image classification tasks [46,47], is used for the feature network of cGAN-UC as well as the deterministic model. The architectures of cGAN-UC and DenseNet used in this experiment are provided in Section C.2 and C.3 in the Supplementary Materials. As a result, the estimation accuracy of the two models is marginally different, where the test set prediction accuracy of cGAN-UC and ordinary DenseNet is 94.4% and 94.1%, respectively, which corresponds to a 0.3% point of enhancement, by using cGAN-UC.

Moreover, it is demonstrated that there exists a significant performance difference in the uncertainty measures of the models. In short, the conventional method to estimate the uncertainty does not perform well in general. Specifically, there is a sort of overfitting in the deterministic model where the model has a high certainty for wrong answers. For instance, in the test set of CIFAR-10, the median softmax output of DenseNet for the wrong answers is 0.968 (96.8%), which means that the deterministic classifier is overconfident to the estimations that are actually wrong.

**Figure 4:**
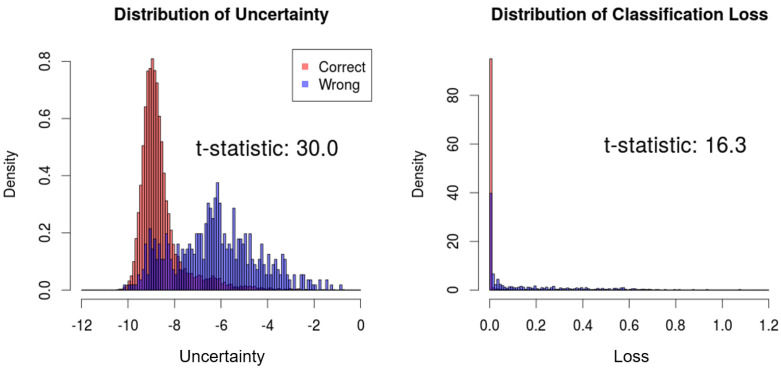
Comparison between the proposed uncertainty and classification loss in the test set of CIFAR-10. Light red indicates the uncertainty/loss distributions of which predictions are correct. Blue indicates the uncertainty/loss distribution of which predictions are wrong.

We compare the distributions of the proposed uncertainty measure and the conventional uncertainty measure with respect to correct and wrong predictions. Then, the *t*-test is performed to measure the difference between the two distributions, which can indicate the correlation between the estimated uncertainty and actual prediction results. As a result, the t-statistics of the proposed uncertainty measure and the classification loss are 30.0 and 16.3, respectively, which signifies superior performance to estimate the uncertainty of predictions (Figure 4).

**Figure 5:**
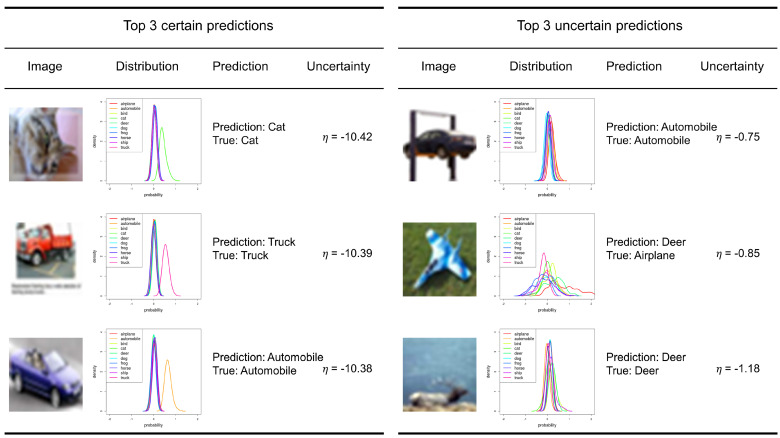
Certain predictions and uncertain predictions for the test set of CIFAR-10. Top 3 certain predictions by cGAN-UC (**left**). Top 3 uncertain predictions by cGAN-UC (**right**). The ’Distribution’ column corresponds to the form of prediction results of cGAN-UC; each color indicates the estimated distribution for each class. Notice that the prediction result in second row of the uncertain predictions is wrong, and the others are correct.

We preform a qualitative analysis for the most certain predictions and the most uncertain predictions of cGAN-UC (Figure 5). We take the top three certain/uncertain predictions for the analysis. As a result, while the image samples of the certain predictions are obvious samples, those of the uncertain predictions correspond to a sort of outlier, i.e., an automobile in the air with a door, a toy airplane on the grass, and a deer that is hard to recognize. Moreover, the airplane is incorrectly classified as ‘Deer’. The overall results indicate that the proposed framework not only shows superior prediction performance but also can properly measure the uncertainty of the predictions.

### 4.3. Noisy Image Classification with Uncertainty

In this section, we further evaluate the proposed framework with noisy image data since we conjecture that the proposed framework shows robust performance against noise because the adversarial learning process is employed for the training of cGAN-UC. In the adversarial learning process, noisy results that are produced by ordinary samples are rejected by the discriminator; thus, cGAN-UC learns from the rejections, which makes cGAN-UC robust against noise. The proposed framework is evaluated with noisy CIFAR-10 image data, which are obtained by the following:

(17) $$\begin{array}{c} X_{N_{\left(a\right)}}=\left(1-a\right)\cdot X+a\cdot N,\\ N∼\mathrm{Unif}\left(\left[0,1\right],I\left(p\right)\right), \end{array}$$

where *a* indicates a parameter for noise, and $X_{N_{\left(a\right)}}\in R^{p}$ denotes a noisy sample. In short, in each experiment, we take different *a* and then evaluate and compare the classification accuracy of the models. The neural network architectures and the other conditions are the same as those of the previous experiment.

**Figure 6:**
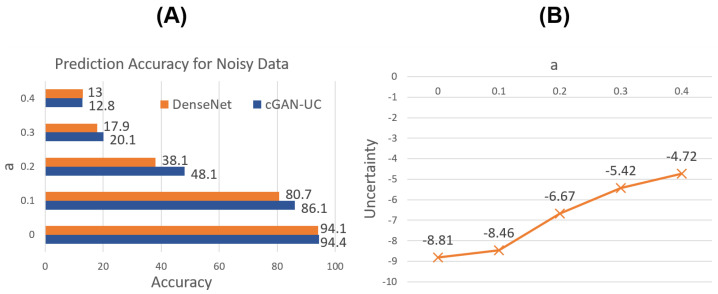
Prediction results for noisy test set of CIFAR-10. (**A**) Prediction accuracy of DenseNet and cGAN-UC with respect to noise. (**B**) The proposed uncertainty measure in cGAN-UC with respect to noise.

Figure 6A shows the prediction results for the noisy data. As expected, for not only the original image data but also the noisy data, it is demonstrated that cGAN-UC outperforms the ordinary DenseNet; moreover, the performance difference is more significant in the noisy data. Specifically, with $X_{N_{\left(0.2\right)}}$, the performance difference is 10.0% point where the accuracy of cGAN-UC and DenseNet is 48.1% and 38.1%, respectively.

In addition, the median of the proposed uncertainty measure is compared with regard to *a*. As shown in Figure 6B, the uncertainty increases as the proportion of noises increases. Such a result also strongly supports the claim that the proposed uncertainty measure actually represents the unsureness of predictions.

**Figure 7:**
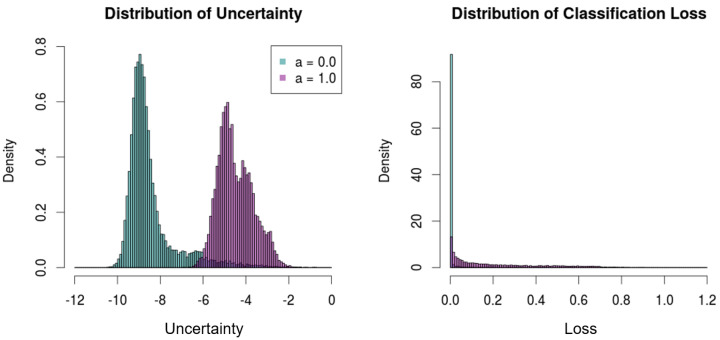
Distributions of the uncertainty and the classification loss with the test set (a=0.0) and noises (a=1.0).

Furthermore, in this experiment, the proposed framework is evaluated with complete noise samples, i.e., $X_{N(1)}$, in order to demonstrate that the proposed method can properly discriminate the meaningless predictions with noises (Figure 7). The deterministic model, DenseNet, however, shows a high certainty for the noises. Specifically, the median softmax output of DenseNet for the noises is 0.875 (87.5%), which reflects the limitation of deterministic models that cannot describe ‘I don’t know’, as aforementioned.

By contrast, the proposed framework shows low certainty/high uncertainty for noises compared to the results with the test set of CIFAR-10. Figure 7 illustrates the uncertainty distributions of the original test set and the noises. The two distributions are significantly distinct from each other, where the median uncertainty measures of the test set and the noises are −8.81 and −4.63, respectively.

**Figure 8:**
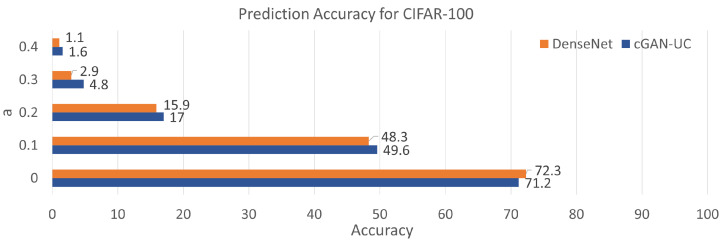
Prediction accuracy for the test set of CIFAR-100. The *a* indicates a noise parameter.

Additionally, it is also demonstrated that the proposed framework can be applied to other datasets (Figure 8). In this experiment, We evaluate the proposed framework with another image dataset called CIFAR-100, which contains 50,000 image samples with 100 different categories in the training set. The image sizes and the number of test samples are the same with CIFAR-10. In the evaluation with CIFAR-100, although the prediction accuracy of the proposed model is slightly inferior to the deterministic model when the models are evaluated with complete data, cGAN-UC outperforms DenseNet with all the scenarios using noisy data, similar to the results of CIFAR-10. Such a result also indicates the noise-resistant feature and generality of the proposed framework. While the proposed model demonstrates a lower performance than DenseNet, we believe that such a performance can be enhanced by increasing the number of sampling process to make the distributions of predictions precise. Since there are 100 categories in CIFAR-100, it is expected that the increase in sampling process can enhance the prediction performance. Such a relationship between number of sampling and performance should be studied further in future work.

## 5. Conclusions

We proposed a predictive probabilistic neural network framework, which corresponds to a different manner of using the generator in cGAN that is initially introduced for sample generation. While cGAN has commonly been employed for conditional sample generation, with extensive experiments in this paper, it is demonstrated that the model also can be used as a predictive model. In addition, we introduced the uncertainty measures for prediction results of the proposed framework. The uncertainty of prediction is calculated by the entropy and relative entropy for regression problems and classification problems, respectively. The proposed framework was evaluated with stock market data and an image classification task. As a result, the proposed framework demonstrates superior prediction performance and successfully estimates the uncertainty of predictions.

Moreover, interestingly, the proposed framework showed robust performances for noisy data, compared to the deterministic model. We conjecture that these results are due to the adversarial learning process of the proposed framework, where noisy outcomes are rejected by the discriminator, and then the generator learns from the failures. For noisy data, since the performance gain by using the proposed framework is significant, such properties should be investigated further for future work.

In addition, for additional experiments, the proposed framework is evaluated with interpolations and other conditions with different degree of noises and shows superior performance as well. The full results for these additional experiments are provided in Section A and B in the Supplementary Materials. We expect that the proposed framework can be a significant breakthrough in predictive neural network model since the proposed framework can predict the uncertainty of estimation that the conventional neural networks can hardly perform and has a possibility to produce superior performance for prediction tasks. Moreover, due to the recent developments in GAN training, the proposed framework, compared to BNNs, has advantages in adopting deep architectures and convergence in training process, which have been constant issues in using probabilistic neural networks.

## Figures and Tables

**Table 1:** Correlation coefficients in the test set. The notation MODEL-n denotes n-layer MODEL. The cGAN-UC indicates the proposed framework, i.e., cGAN for estimation with uncertainty. The means and the standard deviations are obtained over 5 repetitions. In each metric, the best performance is indicated in bold.

Methods	Point Estimation vs. Target	Uncertainty vs. Error
Ridge regression	0.055	N/A *
Lasso regression	0.047	N/A *
Random forest	0.050 ± 0.002	N/A *
ANN-3	0.065 ± 0.019	N/A *
ANN-5	0.065 ± 0.021	N/A *
ANN-7	0.052 ± 0.032	N/A *
BNN-3	0.021 ± 0.019	0.102 ± 0.081
BNN-5	N/A †	N/A †
cGAN-UC-3	0.076 ± 0.025	0.259 ± 0.031
cGAN-UC-5	**0.084** ± **0.052**	0.278 ± 0.021
cGAN-UC-7	0.056 ± 0.076	**0.315** ± **0.061**

* The uncertainty cannot be calculated with the deterministic models. † The model failed to converge within 5000 epochs.

## Data Availability

The Python code used in this paper is partially available in https://github.com/BrainJellyPie/cGANUC (accessed on 14 September 2021).

## Supplementary Materials

The following are available online at https://www.mdpi.com/article/10.3390/s21186194/s1, Supplementary Materials.

## References

[1] Hu J., Shen L., Sun G. Squeeze-and-excitation networks. Proceedings of the IEEE Conference on Computer Vision and Pattern Recognition (CVPR).

[2] Zoph B., Vasudevan V., Shlens J., Le Q.V. Learning transferable architectures for scalable image recognition. Proceedings of the IEEE Conference on Computer Vision and Pattern Recognition (CVPR).

[3] Xie S., Kirillov A., Girshick R., He K. (2019). Exploring Randomly Wired Neural Networks for Image Recognition. arXiv.

[4] Lopez R., Regier J., Cole M.B., Jordan M.I., Yosef N. (2018). Deep generative modeling for single-cell transcriptomics. Nat. Methods.

[5] Liu C., Chen L.C., Schroff F., Adam H., Hua W., Yuille A., Fei-Fei L. Auto-deeplab: Hierarchical neural architecture search for semantic image segmentation. Proceedings of the IEEE Conference on Computer Vision and Pattern Recognition (CVPR).

[6] Lin Y.J., Chao T.K., Khalil M.A., Lee Y.C., Hong D.Z., Wu J.J., Wang C.W. (2021). Deep Learning Fast Screening Approach on Cytological Whole Slides for Thyroid Cancer Diagnosis. Cancers.

[7] Murchan P., Ó’Brien C., O’Connell S., McNevin C.S., Baird A.M., Sheils O., Ó Broin P., Finn S.P. (2021). Deep Learning of Histopathological Features for the Prediction of Tumour Molecular Genetics. Diagnostics.

[8] Desportes L., Fijalkow I., Andry P. (2021). Deep Reinforcement Learning for Hybrid Energy Storage Systems: Balancing Lead and Hydrogen Storage. Energies.

[9] Suri J.S., Agarwal S., Pathak R., Ketireddy V., Columbu M., Saba L., Gupta S.K., Faa G., Singh I.M., Turk M. (2021). COVLIAS 1.0: Lung Segmentation in COVID-19 Computed Tomography Scans Using Hybrid Deep Learning Artificial Intelligence Models. Diagnostics.

[10] Stefano A., Comelli A. (2021). Customized Efficient Neural Network for COVID-19 Infected Region Identification in CT Images. J. Imaging.

[11] Blundell C., Cornebise J., Kavukcuoglu K., Wierstra D. Weight Uncertainty in Neural Network. Proceedings of the International Conference on Machine Learning (ICML).

[12] Gal Y., Ghahramani Z. Dropout as a Bayesian Approximation: Representing Model Uncertainty in Deep Learning. Proceedings of the International Conference on Machine Learning (ICML).

[13] Kendall A., Gal Y. What uncertainties do we need in bayesian deep learning for computer vision?. Proceedings of the Advances in Neural Information Processing Systems (NeurIPS).

[14] Brock A., Donahue J., Simonyan K. Large scale gan training for high fidelity natural image synthesis. Proceedings of the International Conference on Learning Representations (ICLR).

[15] Karras T., Aila T., Laine S., Lehtinen J. Progressive growing of gans for improved quality, stability, and variation. Proceedings of the International Conference on Learning Representations (ICLR).

[16] Miyato T., Kataoka T., Koyama M., Yoshida Y. Spectral normalization for generative adversarial networks. Proceedings of the International Conference on Learning Representations (ICLR).

[17] Lee M., Seok J. (2020). Score-Guided Generative Adversarial Networks. arXiv.

[18] Lee M., Tae D., Choi J.H., Jung H.Y., Seok J. (2020). Improved Recurrent Generative Adversarial Networks with Regularization Techniques and a Controllable Framework. Inf. Sci..

[19] Miyato T., Koyama M. cGANs with projection discriminator. Proceedings of the International Conference on Learning Representations (ICLR).

[20] Zhang H., Goodfellow I., Metaxas D., Odena A. (2018). Self-attention generative adversarial networks. arXiv.

[21] Lee M., Seok J. (2019). Controllable generative adversarial network. IEEE Access.

[22] Odena A., Buckman J., Olsson C., Brown T.B., Olah C., Raffel C., Goodfellow I. Is generator conditioning causally related to gan performance? In Proceedings of the International Conference on Machine Learning (ICML), Stockholm, Sweden, 10–15 July 2018.

[23] Liu Q., Liu W., Yao J., Liu Y., Pan M. (2021). An Improved Method of Reservoir Facies Modeling Based on Generative Adversarial Networks. Energies.

[24] Yang H.D. (2021). Restoring Raindrops Using Attentive Generative Adversarial Networks. Appl. Sci..

[25] Su Y.H., Jiang W., Chitrakar D., Huang K., Peng H., Hannaford B. (2021). Local Style Preservation in Improved GAN-Driven Synthetic Image Generation for Endoscopic Tool Segmentation. Sensors.

[26] Hassani H., Razavi-Far R., Saif M., Palade V. (2021). Generative Adversarial Network-Based Scheme for Diagnosing Faults in Cyber-Physical Power Systems. Sensors.

[27] Wang H., Wang J., Bai K., Sun Y. (2021). Centered Multi-Task Generative Adversarial Network for Small Object Detection. Sensors.

[28] Lin M., Liu L., Wang F., Li J., Pan J. (2021). License Plate Image Reconstruction Based on Generative Adversarial Networks. Remote Sens..

[29] Al-Shargabi A.A., Alshobaili J.F., Alabdulatif A., Alrobah N. (2021). COVID-CGAN: Efficient Deep Learning Approach for COVID-19 Detection Based on CXR Images Using Conditional GANs. Appl. Sci..

[30] Sun S., Chen C., Carin L. Learning structured weight uncertainty in bayesian neural networks. Proceedings of the 20th International Conference on Artificial Intelligence and Statistics, AISTATS 2017.

[31] Li C., Stevens A., Chen C., Pu Y., Gan Z., Carin L. Learning weight uncertainty with stochastic gradient mcmc for shape classification. Proceedings of the IEEE Conference on Computer Vision and Pattern Recognition (CVPR).

[32] Teye M., Azizpour H., Smith K. Bayesian Uncertainty Estimation for Batch Normalized Deep Networks. Proceedings of the International Conference on Machine Learning (ICML).

[33] Creswell A., White T., Dumoulin V., Arulkumaran K., Sengupta B., Bharath A.A. (2018). Generative adversarial networks: An overview. IEEE Signal Process. Mag..

[34] Ghosh A., Kulharia V., Namboodiri V.P., Torr P.H., Dokania P.K. Multi-agent diverse generative adversarial networks. Proceedings of the IEEE Conference on Computer Vision and Pattern Recognition (CVPR).

[35] Xu T., Zhang P., Huang Q., Zhang H., Gan Z., Huang X., He X. Attngan: Fine-grained text to image generation with attentional generative adversarial networks. Proceedings of the IEEE Conference on Computer Vision and Pattern Recognition (CVPR).

[36] Saito Y., Takamichi S., Saruwatari H. (2018). Statistical parametric speech synthesis incorporating generative adversarial networks. IEEE/ACM Trans. Audio Speech Lang. Process..

[37] Goodfellow I., Pouget-Abadie J., Mirza M., Xu B., Warde-Farley D., Ozair S., Courville A., Bengio Y. Generative adversarial nets. Proceedings of the Advances in Neural Information Processing Systems (NeurIPS).

[38] Mirza M., Osindero S. (2014). Conditional generative adversarial nets. arXiv.

[39] Antipov G., Baccouche M., Dugelay J.L. Face aging with conditional generative adversarial networks. Proceedings of the IEEE International Conference on Image Processing (ICIP).

[40] Liu Y., Qin Z., Wan T., Luo Z. (2018). Auto-painter: Cartoon image generation from sketch by using conditional Wasserstein generative adversarial networks. Neurocomputing.

[41] Bessler W., Opfer H., Wolff D. (2017). Multi-asset portfolio optimization and out-of-sample performance: An evaluation of Black–Litterman, mean-variance, and naïve diversification approaches. Eur. J. Financ..

[42] Pedersen J.L., Peskir G. (2017). Optimal mean-variance portfolio selection. Math. Financ. Econ..

[43] Laengle S., Loyola G., Merigo J.M. (2017). Mean-variance portfolio selection with the ordered weighted average. IEEE Trans. Fuzzy Syst..

[44] Yun H., Lee M., Kang Y.S., Seok J. (2020). Portfolio management via two-stage deep learning with a joint cost. Expert Syst. Appl..

[45] Lee M., Seok J. (2020). Regularization Methods for Generative Adversarial Networks: An Overview of Recent Studies. arXiv.

[46] Huang G., Liu S., Van der Maaten L., Weinberger K.Q. Condensenet: An efficient densenet using learned group convolutions. Proceedings of the IEEE Conference on Computer Vision and Pattern Recognition (CVPR).

[47] Huang G., Liu Z., Van Der Maaten L., Weinberger K.Q. Densely connected convolutional networks. Proceedings of the IEEE Conference on Computer Vision and Pattern Recognition (CVPR).

