# Supplementary material for: Estimation with Uncertainty via Conditional Generative Adversarial Networks
Source: Sensors (Basel). 2021 Sep 15;21(18):6194. doi: 10.3390/s21186194 (PMC8471214; doi:10.3390/s21186194)
Supplement: Supplementary file 1 [file sensors-21-06194-s001.zip › sensors-1349164-supplementary.pdf]

Supplementary Materials

A. Additional Experiments for Noisy Data

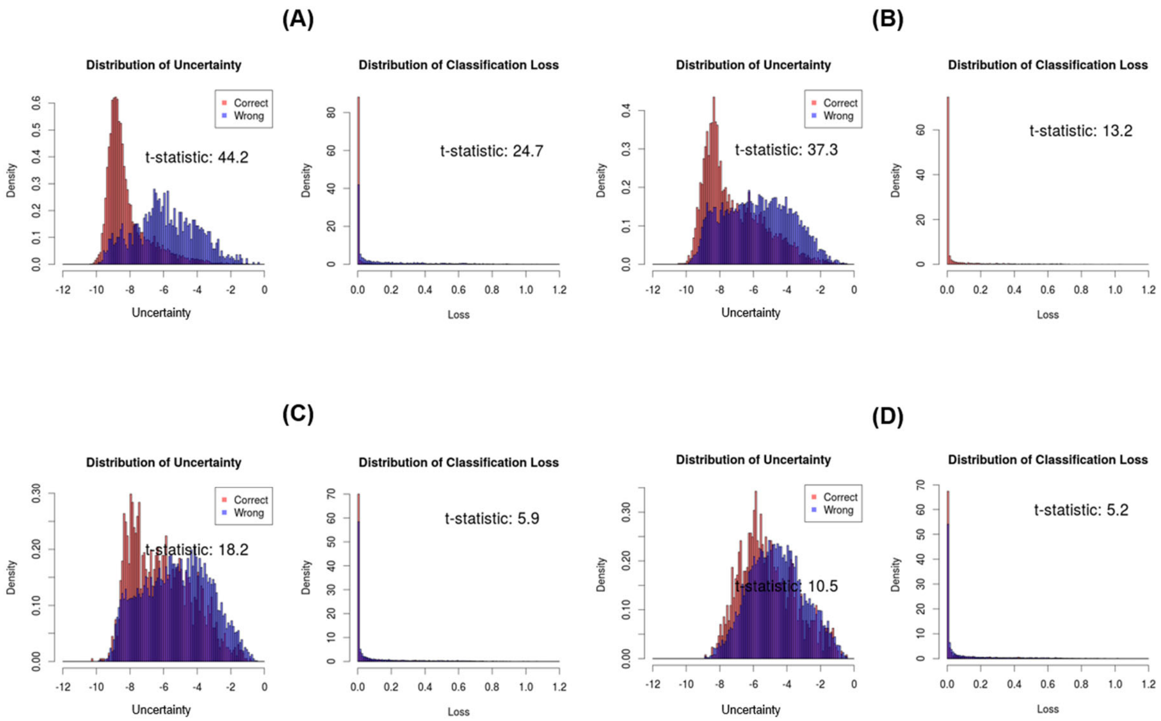

Figure S1. Comparison between the proposed uncertainty and classification loss in the test set with different noise parameter: (A) $a = 0.1$; **(B)** $a = 0.2$; **(C)** $a = 0.3$; **(D)** $a = 0.4$. Light red indicates the distributions of which predictions are correct. Blue indicates the distribution of which predictions are wrong.

In addition, the measured uncertainty is correlated with prediction outcomes (correct/wrong) with the noisy data as well, as shown in Figure A1. The t-statistic for the uncertainty distributions of correct answers and wrong answers is 10.5 with $\boldsymbol{X}_{N(0.4)}$, which corresponds to a p-value of $< 10^{-24}$ that indicates the proposed uncertainty measure performs properly under the challenging condition. Furthermore, the proposed uncertainty measure constantly outperforms the classification loss regardless of a.

B. Additional Experiments with Interpolation

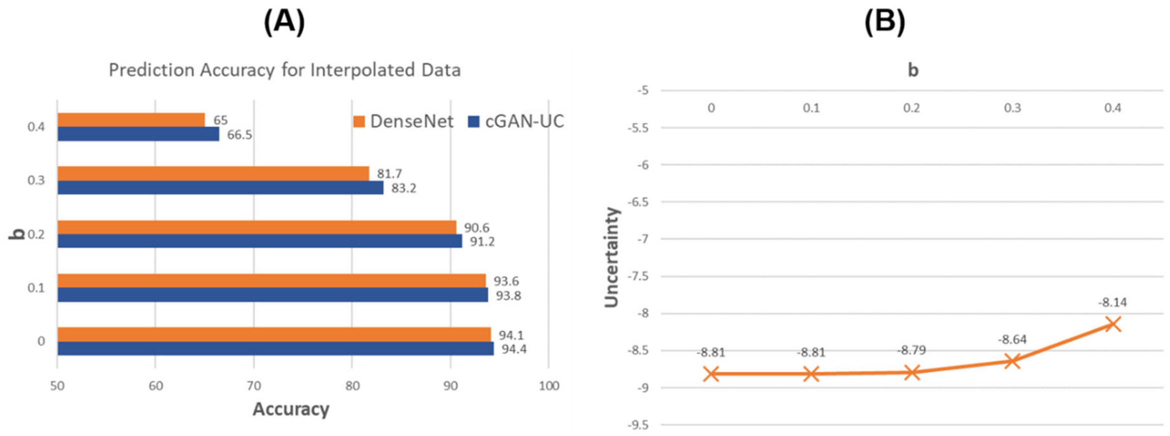

Figure S2. Results for interpolated data. (A) Prediction accuracy; **(B)** Uncertainty with regard to *b*.

In this appendix, cGAN-UC is evaluated with interpolation of samples. The interpolated samples are obtained as follows:

$$\boldsymbol{X}_{inter(i,b)} = (1-b)\cdot\boldsymbol{X}_{(i)} + b\cdot\boldsymbol{X}_{(j)},$$

where $b = (0, 0.5)$ is a parameter for the interpolation, i and j are an index for samples, and $\boldsymbol{X}_{inter(i,b)}$ indicates an interpolated sample. In this experiment, we assume that the true label for $\boldsymbol{X}_{inter(i,b)}$ is same as that of $\boldsymbol{X}_{(i)}$, i.e., $\boldsymbol{Y}\left(\boldsymbol{X}_{inter(i,b)}\right) = \boldsymbol{Y}\left(\boldsymbol{X}_{(i)}\right)$, since $b = (0, 0.5)$. As a result, cGAN-UC constantly outperforms DenseNet regardless of *b*. Also, the uncertainty generally increases with high values of *b*.

C. Neural Networks Architectures

C.1 Neural network architectures of ANNs and BNNs

Table S1. Architecture of ANN-*n*. The r indicates dropout rate. FC(k) indicates a fully connected layer with k number of nodes.

Description	Layers	Num. of blocks
Input	Input layer	$\times 1$
Fully connected<br>Activation<br>Dropout	FC(128)<br>ReLU<br>Dropout(r = 0.2)	$\times (n-2)$
Fully connected<br>Activation	FC(128)<br>ReLU	$\times 1$
Fully connected	FC(1)	$\times 1$

Table S2. Architecture of BNN-*n*. VFC(k) indicates a variational fully connected layer with k number of nodes.

Description	Layers	Num. of blocks
Input	Input layer	$\times 1$
Variational<br>Fully connected<br>Activation	VFC(128)<br>ReLU	$\times (n-2)$
Variational<br>Fully connected<br>Activation	VFC(128)<br>ReLU	$\times 1$
Variational<br>Fully connected	VFC(5)	$\times 1$

C.2 Neural network architectures of DenseNet for CIFAR-10

Table S3. Architecture of DenseNet for CIFAR-10. Conv(*k*) indicates a convolutional layer with *k* number of filters, and FC(*k*) indicates a fully connected layer with *k* number of nodes.

Description	Layers	Num. of blocks
Input	Input layer	× 1
Convolutional	Conv(64, filter size=(5,5), stride=1)	
Batch-normalization	BN(momentum=0.99)	× 1
Activation	ReLU	
Batch-normalization	BN(momentum=0.99)	
Activation	ReLU	
Convolutional	Conv(128, filter size=(1,1), stride=1)	
Batch-normalization	BN(momentum=0.99)	× 6
Activation	ReLU	
Convolutional	Conv(32, filter size=(3,3), stride=1)	
Concatenate	Concat(previous, block start)	
Batch-normalization	BN(momentum=0.99)	
Activation	ReLU	× 1
Convolutional	Conv((block start)*0.5, filter size=(1,1), stride=1)	
Average pooling	AvgPool(filter size=(2,2), stride=2)	
Batch-normalization	BN(momentum=0.99)	
Activation	ReLU	
Convolutional	Conv(128, filter size=(1,1), stride=1)	
Batch-normalization	BN(momentum=0.99)	× 12
Activation	ReLU	
Convolutional	Conv(32, filter size=(3,3), stride=1)	
Concatenate	Concat(previous, block start)	
Batch-normalization	BN(momentum=0.99)	
Activation	ReLU	× 1
Convolutional	Conv((block start)*0.5, filter size=(1,1), stride=1)	
Average pooling	AvgPool(filter size=(2,2), stride=2)	
Batch-normalization	BN(momentum=0.99)	
Activation	ReLU	
Convolutional	Conv(128, filter size=(1,1), stride=1)	
Batch-normalization	BN(momentum=0.99)	× 24
Activation	ReLU	
Convolutional	Conv(32, filter size=(3,3), stride=1)	
Concatenate	Concat(previous, block start)	
Batch-normalization	BN(momentum=0.99)	
Activation	ReLU	× 1
Convolutional	Conv((block start)*0.5, filter size=(1,1), stride=1)	
Average pooling	AvgPool(filter size=(2,2), stride=2)	
Batch-normalization	BN(momentum=0.99)	
Activation	ReLU	
Convolutional	Conv(128, filter size=(1,1), stride=1)	
Batch-normalization	BN(momentum=0.99)	× 16
Activation	ReLU	
Convolutional	Conv(32, filter size=(3,3), stride=1)	
Concatenate	Concat(previous, block start)	
Batch-normalization	BN(momentum=0.99)	
Activation	ReLU	× 1
Global average pooling	GlobAvgPool *	
Fully connected	FC(10)	

* The output of the global average pooling is used for the feature network of cGAN-UC

C.3 Neural network architectures of cGAN-UC for CIFAR-10

Table S4. Architecture of the generator of cGAN-UC for CIFAR-10. FC(k) indicates a fully connected layer with k number of nodes.

Description	Layers	Feature encoding
Input	Input layer($\mathbf{Z} \sim N(\mathbf{0},\mathbf{I}(8))$)	
Feature input *		Feature input layer($\mathcal{M}_F(\boldsymbol{X};\boldsymbol{\theta}_F)$)
Fully connected	FC(16)	
Feature encoding		FC(16)
Projection	Element-wise multiply	
Fully connected	FC(64)	
Activation	ReLU	
Feature encoding		FC(64)
Projection	Element-wise multiply	
Fully connected	FC(128)	
Activation	ReLU	
Feature encoding		FC(128)
Projection	Element-wise multiply	
Fully connected	FC(10)	

* The output of the global average pooling layer of DenseNet is used for the feature input

Table S5. Architecture of the discriminator of cGAN-UC for CIFAR-10. FC(k) indicates a fully connected layer with k number of nodes.

Description	Layers	Feature encoding
Input	Input layer($\boldsymbol{Y}$)	
Feature input *		Feature input layer($\mathcal{M}_F(\boldsymbol{X};\boldsymbol{\theta}_F)$)
Fully connected	FC(128)	
Feature encoding		FC(128)
Projection	Element-wise multiply	
Fully connected	FC(1)	

* The output of the global average pooling layer of DenseNet is used for the feature input

C.4 Neural network architectures of cGAN-UC for stock market data

Table S6. Architecture of the feature network of cGAN-UC for stock market data. FC(k) indicates a fully connected layer with k number of nodes.

Description	Layers	Num. of blocks
Input	Input layer	× 1
Fully connected	FC(128)	× 3
Activation	ReLU	
Dropout	Dropout(r = 0.2)	
Batch-normalization	BN(momentum=0.99)	
Fully connected *	FC(128)	× 1
Fully connected	FC(5)	× 1

* The output is used for the feature network of cGAN-UC

Table S7. Architecture of the generator of cGAN-UC-*n* for stock market data. FC(k) indicates a fully connected layer with k number of nodes.

Description	Layers	Feature encoding	Num. of blocks
Input	Input layer($\mathbf{Z} \sim N(\mathbf{0}, \mathbf{I}(4))$)		× 1
Feature input *		Feature input layer($\mathcal{M}_F(\boldsymbol{X}; \boldsymbol{\theta}_F)$)	
Fully connected	FC(4)		× 1
Feature encoding		FC(4)	
Projection	Element-wise multiply		
Fully connected	FC(128)		$\times (n-1)$
Activation	ReLU		
Fully connected	FC(5)		× 1

* The output of the feature network is used for the feature input

Table S8. Architecture of the discriminator of cGAN-UC-*n* for stock market data. FC(k) indicates a fully connected layer with k number of nodes.

Description	Layers	Feature encoding	Num. of blocks
Input	Input layer($\boldsymbol{Y}$)		× 1
Feature input *		Feature input layer($\mathcal{M}_F(\boldsymbol{X}; \boldsymbol{\theta}_F)$)	
Fully connected	FC(128)		$\times (n-1)$
Activation	ReLU		
Fully connected	FC(4)		× 1
Feature encoding		FC(4)	
Projection	Element-wise multiply		
Fully connected	FC(1)		× 1

* The output of the feature network is used for the feature input

D. The training process for cGAN-UCs

Conventional training techniques for generative adversarial networks are employed to train cGAN-UCs. Specifically, the hinge loss for the discriminator and Wasserstein distance [1].

Before training cGAN-UCs, the feature networks are trained, and weights of the feature networks are fixed during the training of cGAN-UC. The architecture of the feature networks for stock market data and CIFAR-10 are provided in Table A3 and A6, respectively. The feature networks aim to estimate the labels $\boldsymbol{Y}(\boldsymbol{X})$, during the training process, then, feature outputs, i.e., the penultimate layer of the networks, are used for cGAN-UCs as the feature inputs. The training of the feature networks is conducted over 5,000 epochs and 200 epochs for the stock market data and CIFAR-10, respectively. The adam optimizer with a learning rate of 0.0001 is used. Batch size is set at 128.

The training of cGAN-UCs is conducted by the adam optimizer with different learning rates for the discriminator and the generator, which are set at 0.0008 and 0.0002, conventional values for GANs [2-4]. Batch size is set at 512 and 1,024 for the stock market data and CIFAR-10, respectively. The training is conducted over 10,000 epochs for CIFAR-10.

E. Preprocessing Method for Stock Market Data

The daily close price data of NASDAQ-100 are preprocessed for the training of regression models used for the experiment. Specifically, returns are used for the inputs and the target of the models, which are calculated as follows:

$$r_{k,c} = \frac{p_c - p_k}{p_c},$$

where $c = \{31, \dots, n-5\}$, n is the number of price samples in the training set, p denotes the price, $k = \{c-1, \dots, c-30\} \cup \{c+5\}$, and $r_{k,c}$ indicates the return data.

We use $r_{k,c}$ in which $k = \{c-1, \dots, c-30\}$ as the inputs and $r_{k,c}$ in which $k = c+5$ as the target for the training of the models, which means the models predict 5-day returns by using a sequence of the returns of the past 30 days.

References

[1] T. Miyato and M. Koyama, “cgans with projection discriminator,” International Conference on Learning Representations (ICLR), 2018.

[2] A. Brock, J. Donahue, and K. Simonyan, “Large scale gan training for high fidelity natural image synthesis,” International Conference on Learning Representations (ICLR), 2019.

[3] H. Zhang, I. Goodfellow, D. Metaxas, and A. Odena, “Self-attention generative adversarial networks,” arXiv preprint, 2018.

[4] M. Lee and J. Seok, “Controllable generative adversarial network,” IEEE Access, vol. 7, pp. 28158–28169, 2019.
